# To Achieve an Earlier IFN-γ Response Is Not Sufficient to Control *Mycobacterium tuberculosis* Infection in Mice

**DOI:** 10.1371/journal.pone.0100830

**Published:** 2014-06-24

**Authors:** Cristina Vilaplana, Clara Prats, Elena Marzo, Carles Barril, Marina Vegué, Jorge Diaz, Joaquim Valls, Daniel López, Pere-Joan Cardona

**Affiliations:** 1 Unitat de Tuberculosi Experimental (UTE), Fundació Institut d'Investigació en Ciències de la Salut Germans Trias i Pujol, Universitat Autònoma de Barcelona, CIBERES, Badalona, Catalonia, Spain; 2 Escola Superior d'Agricultura de Barcelona, Departament de Física i Enginyeria Nuclear, Universitat Politècnica de Catalunya, C/Esteve Terradas, Castelldefels, Catalonia, Spain; Johns Hopkins University School of Medicine, United States of America

## Abstract

The temporo-spatial relationship between the three organs (lung, spleen and lymph node) involved during the initial stages of *Mycobacterium tuberculosis* infection has been poorly studied. As such, we performed an experimental study to evaluate the bacillary load in each organ after aerosol or intravenous infection and developed a mathematical approach using the data obtained in order to extract conclusions. The results showed that higher bacillary doses result in an earlier IFN-γ response, that a certain bacillary load (BL) needs to be reached to trigger the IFN-γ response, and that control of the BL is not immediate after onset of the IFN-γ response, which might be a consequence of the spatial dimension. This study may have an important impact when it comes to designing new vaccine candidates as it suggests that triggering an earlier IFN-γ response might not guarantee good infection control, and therefore that additional properties should be considered for these candidates.

## Introduction

The ability to subvert the host's immune system by blocking its ability to detect the pathogen is considered to be one of the main characteristics of *Mycobacterium tuberculosis* infection [1]. Different authors have elaborated several hypotheses to explain this phenomenon, most of which focus on the ability of *M. tuberculosis* to avoid apoptosis [2] and block antigen presentation [3], [4] or lysosomal degradation [5], amongst others. However, these hypotheses are based on mechanisms studied at a molecular level, thus meaning that the holistic point of view has been somewhat overlooked [6]. Indeed, the role of lymphoid organs as the targets of infection, the blood and lymphatic circulation, or the intrinsic anatomy of the lung (the key organ in this infection) have been little studied or ignored.

SChackerian [7] and Wolf [8] arrived at the same conclusion, namely that the most important event for achieving early control is fast infection of the hilar lymph node (as production of specific lymphocytes is induced earlier), using two different experimental models of TB (in mice and macaques). In contrast, Bru and Cardona [9] arrived at a slightly different conclusion using virtual models, namely that although the time at which the immune response is triggered is important, the local production of chemokines which can attract those lymphocytes to activate the infected macrophages is equally important. This supported the idea of granuloma formation being necessary as a tool for controlling the infection by making the infectious focus “visible” to the specific lymphocytes [10], in contrast to the proposal that the granuloma could be a “foe” by allowing the bacilli to persist [11]. Observations from other researchers [Ian Orme, personal communication] and our group [9], [12] also support the beneficial role of the granuloma. Thus, no lesions can be seen in the lung during the first two weeks of a low dose aerosol infection despite the global bacillary load (BL) increasing from 10^2^ to 10^5^, thereby indirectly demonstrating a “hidden” initial phase of the infection [6]. This unicellular infection phase appears to last about 10–15 days [9], [13] and coincides with the time need to detect the first granulomas [7], [8], [14]. During this period, *M. tuberculosis* grows inside individual infected macrophages that cannot be detected by specific lymphocytes, probably because they are not able to produce sufficient chemokines to attract them. The existence of this phase could also support previous and recent results claiming the detection of bacilli in healthy tissue [15], [16].

In order to study the temporo-spatial relationship between the three organs (lung, spleen and lymph node) involved during initial *M. tuberculosis* infection, we designed an experimental study in which mice were infected via aerosol (AER) or endovenously (EV) and consequently followed-up, subsequently developing a mathematical approach to reproduce the results obtained experimentally and to extract conclusions.

## Results

### Higher Bacillary Doses Trigger an Earlier IFN-γ Response

For consensus, and taking into account the experimental results, we considered 20 SFU/250,000 cells to be the threshold for onset of the specific IFN-γ response. This value was chosen because, whenever a significant change was detected in the ELISPOT results, it always meant that this value had been exceeded. In the EV model, the immune response was first detected in the spleen in all cases (Table 1). A comparison between the specific responses shows how the immunity mainly targets ESAT-6. At the highest dose (10^5^ CFU/ml), specific lymphocytes are detected first in the lung and then in the lymph node, thus reflecting the accumulation of circulating IFN-γ producing lymphocytes from the spleen before immune specific IFN-γ response is triggered in the lymph nodes. This suggests that some degree of BL is needed for the induction of immunity. In the EV model, higher challenge doses lead to earlier IFN-γ responses.

**Table 1:** Detection of PPD-specific IFN-γ response.

DETECTION PPD-SPECIFIC IFN-γ RESPONSE (in days)			
	EV exp (depending on the inoculum, in CFU)		
ORGAN	10^2^	10^4^	10^5^
spleen	14	8	5
lymph node	20	17	11
lung	≥28	20	8

DETECTION ESAT-6-SPECIFIC IFN-γ RESPONSE (in days)			
	EV exp (depending on the inoculum, in CFU)		
ORGAN	10^2^	10^4^	10^5^
spleen	14	8	5
lymph node	11	14	11
lung	20	17	8

Day of detection of the PPD- and ESAT-6-specific IFN-γ responses in the three different organs obtained for all experiments performed.

In contrast, there is no difference as regards onset of the IFN-γ response among the organs in the aerosol model: it appears at the same time in all of them.

### A Specific Bacillary Load Must Be Reached to Trigger the IFN-γ Response

Considering the minimum time required to trigger the IFN-γ response obtained from the highest challenge model (10^5^), we estimated the delay between this trigger and onset of the IFN-γ response to be 2.5 days. In light of this value, we evaluated the BL threshold required to trigger the IFN-γ response by analysing the level thereof 2.5 days prior to onset of the IFN-γ response in each experimental model. Specifically, in each case we determined which organ had the highest BL 2.5 days before the onset (i.e., which the triggering organ was) and which this threshold value was. The results showed that the BL level required is relatively constant, at around 3.5 log10 (Table 2). This threshold is exceeded from the beginning in the highest dose EV model, thus validating the hypothesis used in these estimations (i.e., that the 10^5^ dose is enough for triggering the IFN-γ response immediately after infection).

**Table 2:** Onset of the specific IFN-γ response.

APPEARANCE OF THE SPECIFIC IFN-γ RESPONSE				
	EV exp (depending on the inoculum, in CFU)			AER exp
	10^2^	10^4^	10^5^	
Organ of appearance of the specific IFN-γ response (>20 SFU) and day of appearance of such response	spleen	spleen	spleen	spleen and lymph node
	11.7±0.4 days	6.4±0.9 days	2.5±0.5 days	16.4±2.3 days
Organ that triggers the specific IFN-γ response and its BL 2.5 days before appearing the specific IFN-γ response	spleen	spleen	spleen	lymph node
	3.41±0.21 log CFU	3.77±0.17 log CFU	4.66±0.14 log CFU	<3.61±0.23 log CFU

First row: estimated onset of the specific IFN-γ response taking into account the organ and day on which it appears. The moment at which the 20 SFU threshold would have been surpassed is linearly interpolated between two consecutive experimental points (last point with SFU<20 and first point with SFU>20). Second row: organ and BL of the organ that would have triggered the IFN-γ response 2.5 days before its estimated appearance (i.e., organ with the highest BL at that moment).

### Control of the BL Is Not Immediate After Onset of the IFN-γ Response

**Figure 1:**
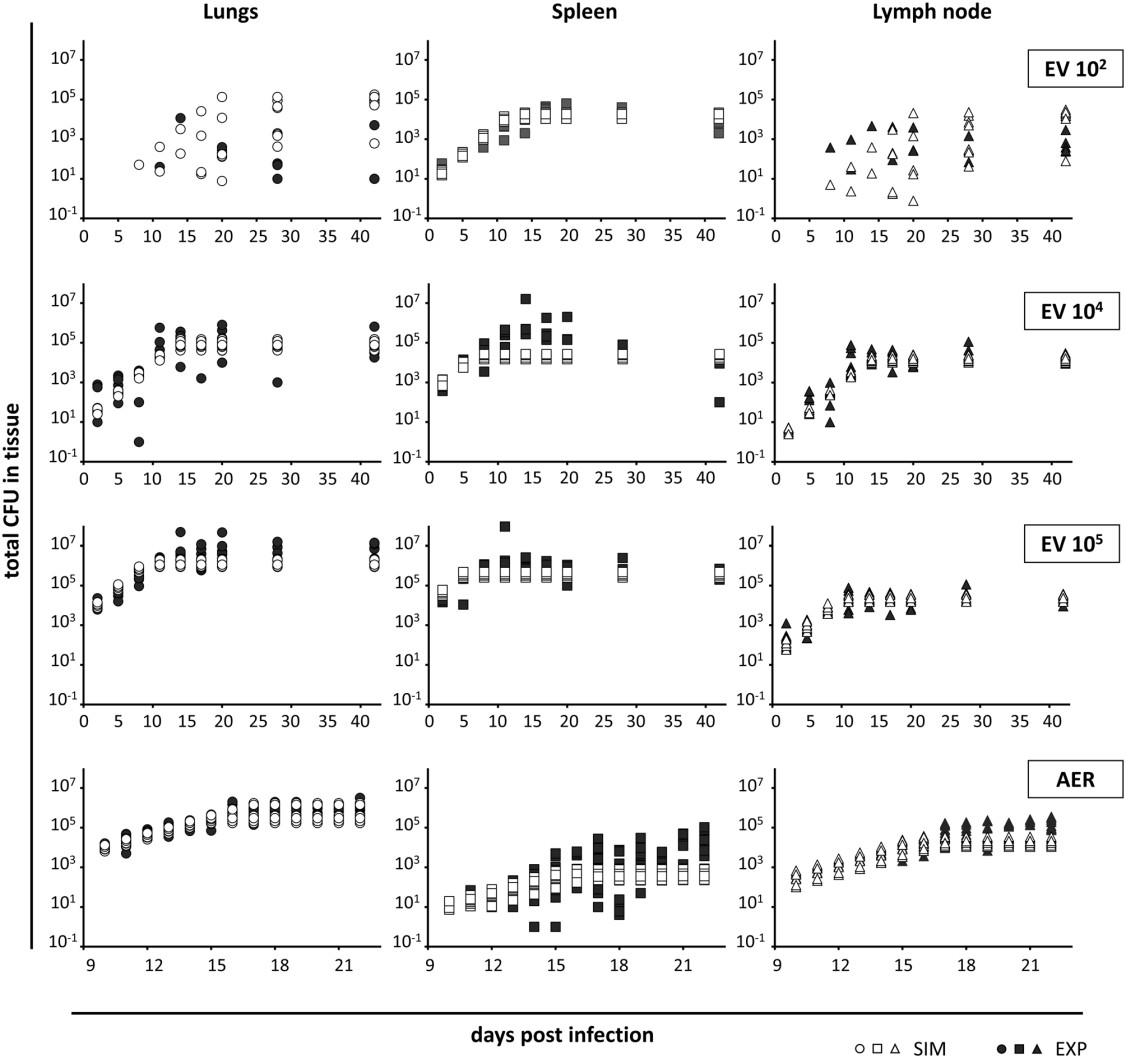
Output of the *in silico* model and the corresponding experimental results. The output of the in silico model (white symbols) are represented considering EV infections of 10^2^, 10^4^ and 10^5^ CFU/mL and an AER infection. The experimental results are represented by black symbols. The results are shown individually for each organ (lungs (circles), spleen (squares) and lymph node (triangles)). Ten repetitions of each simulation were performed by changing the random seed. Each experimental point corresponds to an individual mouse.

Figure 1 shows the evolution of the BL in the different organs as obtained in the experimental *in vivo* models (in black). After the EV challenge, the IFN-γ response was estimated to appear at days 2.5 (initial inoculum  = 10^5^), 6.47 (10^4^) and 11.77 (10^2^) (Table 2); BL control occurs subsequently. The sooner the IFN-γ response is triggered, the sooner BL control occurs, at least in spleen and LN. This effect is not seen in lungs. Table 3 shows the delay between the appearance of the IFN-γ response and the effective control of the BL, per organ.

**Table 3:** Delays between the appearance of the IFN-γ response and the effective control of the BL, per challenge dose and route, and per organ.

	Spleen	Lymph node	Lungs
**EV exp 10^2^**			
Day of estimated appearence of the IFN-γ response ^(1)^	11,7	-	-
Day of detected control of BL	17	17	14
Delay between appearence of the IFN-γ response and BL control (days)	5,3	5,3	2,3
**EV exp 10^4^**			
Day of estimated appearence of the IFN-γ response ^(1)^	6,4	-	-
Day of detected control of BL	14	14	11
Delay between appearence of the IFN-γ response and BL control (days)	7,6	7,6	4,6
**EV exp 10^5^**			
Day of estimated appearence of the IFN-γ response ^(1)^	2,5	-	-
Day of detected control of BL	11	11	14
Delay between appearence of the IFN-γ response and BL control (days)	8,5	8,5	11,5
**AER exp**			
Day of estimated appearence of the IFN-γ response ^(1)^	16,4	-	16,4
Day of detected control of BL	21	21	16
Delay between appearence of the IFN-γ response and BL control (days)	4,6	4,6	≈0

^(1)^ Deviations shown in Table 2

These delays are evaluated by subtraction between the day when BL control is experimentally detected at each organ and the day of the estimated appearance of IFN-γ response.

The BL in an organ does not appear to be crucial as regards the control of bacillary growth, as this occurs over a wide range from 10^5^ to 10^7^ CFU/ml depending on the initial infective dose.

In the aerosol-infected model, BL control in the lung, which is the organ with the highest bulk, appears at the same time as the immune response. However, and as is the case for the EV model, BL control in the spleen is achieved 5 days after the onset of the IFN-γ response (i.e. at day 16.43). This value confirms that, although a low BL in the spleen is able to trigger and attract the IFN-γ response, it is not capable of effectively stopping bacillary growth.

### The Mathematical Model Designed Reproduces the Experimental Data and Highlights the Importance of Considering the Spatial Factor

The *in silico* model built provided results which agreed well with experimental measurements in terms of time and levels (Figure 1). 10^4^ and 10^5^ series were used for parameter estimation (i.e., for fitting the model to experimental data), and 10^2^ and AER series where used for validation of the model. However, BL control was achieved earlier in the mathematical model than in the *in vivo* models under all conditions.

## Discussion

The aim of this study was to evaluate the bacillary load and specific IFN-γ responses in lung, spleen and lymph node, after aerosol or intravenous infection; and to develop a mathematical approach with the data obtained.

To date, a paramount role has been assigned to rapid infection of the lung lymph node as regards onset of the immune response and better infection control in the lung [7], [8]. The results showed that a critical BL has to be reached to trigger the immune responses (at least in terms of IFN-γ responses) and that as highest is the BL the earlier this response is triggered, and yet (paradoxically) it is not sufficient to stop immediately the BL growth.

The dual role of lymph nodes and spleen as providers of effector immune cells and as infectious foci intrigued us, and anatomy is one of the possible answers. An infected macrophage or dendritic cell could reach the trabecular sinuses, thereby initiating infection of the lymph node in an anatomically different location in the cortex or the medullar sinus, where antigens are presented to B and T cells, respectively [17]. If this is the case, local infection in the lymphoid tissues would not be directly controlled by the local IFN-γ response triggered, which will be spilled to the efferent capillary lymph vessels and therefore to pulmonary circulation thus to the right heart and lungs [18], where it is attracted and further captured by pulmonary infectious sites. Likewise, the spleen, which specializes in retaining and destroying old erythrocytes [19], has two compartments: the red pulp, where an infection is more likely to occur, and the white pulp, in which the antigens are presented and specific lymphocyte proliferation induced [19].

SOur initial hypothesis was that this was possible because lymphatic organs could host an infected site while still being able to develop a specific IFN-γ response against this infection (although not benefiting from it) for anatomical reasons. Indeed, the results of our mathematical model support this. Because of the anatomy of lymphatic tissues, the lymphocytes would follow the lymphatic and blood circulation and be further attracted by other infectious foci. In the case of a low dose aerosol at day 21 post-infection, when the bacillary load in the lung is very high, a lymphocyte generated in the spleen will reach the cava vein and then progress further to the right atrium and right ventricle, eventually reaching the lung. At this point, because of the high infection, the lesions are likely to attract this lymphocyte. If this does not occur, it will continue into the left atrium and ventricle to circulate throughout the rest of the body, perhaps being attracted by the infected lymph nodes or even reaching the spleen. This dynamic was validated by the mathematical model.

Interestingly enough, even in this situation it is not clear whether these lymphocytes will be able to activate the infected macrophages, as a pool of attraction (i.e. a local focus of chemokine production) is needed, suggesting the granuloma allows the attraction of specific lymphocytes [10]. Considering that the initial infection usually takes place inside the alveolar macrophage (in the alveolar space), where immunological surveillance is almost impossible, favoring reinfection, the lack of pool of attraction is a problem in terms of infection control in this timeframe [9], [20].

We are aware that the host immune response against tuberculosis involves a complex combination of cytokines, chemokines and signaling molecules other than the IFN-γ responses, and our study was only focused on PPD and ESAT-6 specific IFN-γ responses. However, as these have been widely used as a marker of tuberculosis infection even if its limitations are commonly known and accepted, we did considered that they could fairly be representative of the host immune response for the study purposes. Moreover, the number of animals involved was also relevant for this decision, as to add more essays to explore other would have meant to increase this number in detrimental of ethics. On the other hand, we tried to keep the parameters and premises of the *in silico* model at minimum, in order to keep it as simple as possible.

Another limitation of our study is that it is based on an experimental mice model, and mice are far from humans. However, in terms of anatomy of the organs studied and dynamics of blood circulation, the results obtained in mice can be totally extrapolated to humans, at least those related with the qualitative dynamics. The quantitative results (for instance, the delay between trigger and onset of IFN-γ response, or the BL threshold for such triggering) may slightly change from mice to human, and thus the mathematical model should be specifically parameterized for the second case.

The mathematical model designed to simulate the complex anatomical relationships between the different infected organs reproduces the general dynamics of the experimental results. As in the generation of any other *in silico* model, assumptions have to been made and the parameters chosen, something we did trying to keep it simple and in accord with experimental observations. The only point where it differs from them is on the delay on controlling the BL, as it only takes into account the global BL of each one without considering the local organization of the infected cells, and cannot incorporate the spatial dimension which might be important for attracting specific lymphocytes to each infectious focus. This may be the reason why faster control of the BL is achieved after onset of the IFN-γ response than in the *in vivo* model, therefore showing the importance of this factor.

In conclusion, although lymphoid organs appear to be organized as a two compartment structures, thus favoring infection control in non-lymphoid organs, the the local attraction of specific lymphocytes to the infected sites is crucial as regards controlling the BL in these organs and on the infection control.

Overall, these data suggest that the key to subversion of the immune response by *M. tuberculosis* is not related to the ability of the bacilli to slow its induction. Indeed, our findings show that the speed of the immune response is not related to the degree of infection control as the ability of infected cells to attract specific lymphocytes appears to be the most critical factor as regards benefitting from the immune response, at least in terms of IFN-γ responses.

This might have a conceptual impact when it comes to designing new prophylactic strategies, especially new vaccine candidates as, if they are able to trigger a rapid IFN-γ response (or even -if we hypothesize- a rapid Th1 immune response), will this be sufficient to cope with the infection? The answer to this is probably not, as this enhanced IFN-γ response will not know where to act as no infectious foci will be generating sufficiently strong attractive signals. In light of the results of our study, we therefore suggest that other strategies focusing on therapeutic vaccines or those generating humoral responses, for example, should perhaps be explored.

## Materials and Methods

### Experimental Data

#### Mice

6–8-Week-old female C57BL/6 specific-pathogen-free (*spf*) mice were obtained from Harlan Laboratories (Sant Feliu de Codines, Catalonia, Spain). The animals were shipped under suitable conditions, with the corresponding certificate of health and origin. Upon arrival, the mice were kept under controlled conditions in a P3 high security facility with sterile food and water “ad libitum”.

#### Ethics

All animal procedures were approved and supervised by the Animal Care Committee of the Germans Trias i Pujol University Hospital and by the Department of Environment of the Catalan Government. Mice were weighed and checked every week following a protocol that monitored weight loss, apparent good health (bristled hair and wounded skin) and behaviour (signs of aggressiveness or isolation). Mice were euthanized with isoflurane (inhalation excess) in all cases in order to avoid any suffering.

#### Experimental plan

Two routes of infection were evaluated: the aerosol and the intravenous route. A total of six animals per experimental group were included in each experiment. The AER experiment was run twice, whereas the EV experiment was run only once due to the already high number of mice involved for the three different inoculation doses being evaluated (10^2^, 10^4^ or 10^5^ Colony Forming Units (CFU) per mL).

The animals were sacrificed at established sequential timepoints. Different timepoints were established for AER and the EV exp based on our previous experience with experimental TB infection. In the aerosol experiment, animals were sacrificed on each day from day 10 to 22 post-infection, whereas in the EV experiment mice were sacrificed on days 0 and 2, 5, 8, 11, 14, 17, 20, 28 and 42 post-infection.

#### Infection

*M. tuberculosis* strain H37Rv Pasteur was grown in Proskauer Beck medium. In the AER experiment, animals were placed in the exposure chamber of an airborne infection apparatus (Glas-col Inc., Terre Haute, IN, USA) for infection with a low dose of *M. tuberculosis.* Nebulization provided an approximate uptake of 20–50 bacilli by mice lungs.

In the EV experiment, a total volume of 0.2 mL containing 10^2^, 10^4^ or 10^5^ CFU per mL (depending on the experimental group) was inoculated into the tail vein of each mouse.

#### Evaluation of the infection

The bacillary load (BL) and specific IFN-γ responses measured were the parameters chosen to evaluate the infection. Both parameters were measured in lung, spleen and lymph nodes, which were extracted and mechanically disrupted in order to obtain tissue homogenates. Lymph nodes samples could occasionally not be harvested at the earliest timepoints as they were too small to be readily recognised, therefore no results were recorded in such cases.

The BL was determined by culturing the samples on Middlebrook 7H11 agar plates (Bennex Ltd, Shannon, Ireland) at 37°C for 21 days. Visible colony forming units (CFU) were counted four weeks later, with data being recorded as the log of the total number of CFUs recovered per organ.

Specific cellular IFN-γ responses against the antigens ESAT-6 (Lionex Diagnostics & Therapeutics, Braunschweig, Germany), which was used at a final concentration of 5 µg/mL, and PPD (Statens Serum Institute, Kobenhavn, Denmark), which was used at a final concentration of 10 µg/mL, were measured using an IFN-γ ELISPOT kit (BD Bioscience, San Diego, CA) after plating 250,000 cells from the tissue homogenates. The ELISPOT assay was further developed and read according to the manufacturer's instructions. The results were expressed as IFN-γ secreting cells as Spot Forming Units (SFU) per 250,000 cells.

### The *In Silico* Model

An *in silico* model based on several premises was designed in order to test our hypothesis regarding the *in vivo* dynamics of the infection. It was fitted to and validated with experimental results and subsequently further used to extract some conclusions about how the flows between the different organs influence the infection. In order to build this model, different variables, processes and parameters were taken into account. These are described below (S1).

### Model Variables

Those quantities that vary with time and represent the dynamics of the system were termed variables. According to the experimental measurements, these variables are the BL and the level of IFN-γ response expressed as IR.

BL_l_(t), BL_s_(t) and BL_ln_(t) are the bacillary loads in lung, spleen and lymph nodes at time *t*, respectively, and IR_l_(t), IR_s_(t) and IR_ln_(t) the immune responses in lung, spleen and lymph nodes at the same time *t*, respectively. The dynamics of these variables are governed by the model processes described below. The output of the model should agree with the experimental behavior of these variables.

### Model Processes

The model processes are those that can make the variables change, and are based on assumptions decided by consensus after checking different possibilities and their effects on the model output, that should be in accord with experimental data. Three such processes were considered: local bacillary growth, the trigger for the specific immune response and the flows between the three different organs studied.

#### Local bacillary growth

In order to model the bacillary growth in each organ (lung, spleen and lymph nodes), the effective net growth arising from replication inside macrophages and bacterial death was taken into account. This growth, which is represented by the classic logistic model, is modulated by the IFN-γ response activity (a_IR_) as follows (Eq. 1).
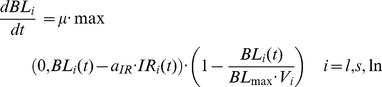
(1)


Thus, Eq. (1) shows the time (t) evolution of BL at each organ (l for lungs, s for spleen and ln for lymph nodes), as modulated by the IFN-γresponse level (IR) in the corresponding organ (i = l, s, ln). The parameters involved are the bacillary effective growth rate (µ), the maximum bacillary load per unit volume (BL_max_), the effective organ volumes (V_i_, i = l, s, ln), and the IFN-γ response activity (a_IR_). The latter represents the number of bacilli that are inactivated per unit of specific IFN-γ response.

#### Bacillary flows between organs

The bacilli grow locally in each organ but can be released into circulation and reach another organ. This is thought to occur after the lysis of infected macrophages, when bacilli are released into the extracellular environment. As such, migration of bacilli from one organ to another is linked to the discrete intervals at which macrophages burst. Consequently, this process is modelled as discrete migrations at certain time intervals corresponding to multiples of T_lysis_ after initial infection of the corresponding organ rather than as a constant flow. The parameter T_lysis_ represents the mean time that a macrophage takes to burst once infected.

The physiological flows (determined by the blood circulation dynamics) important (i.e., numerically relevant to the results) for the dynamics of the model were chosen according to the principle of Occam's razor [21]. That is, all such flows were initially incorporated into the model but only those that were numerically relevant were kept. In the EV case, initial concentrations in the spleen and lungs were similar. If lungs were not initially infected (low concentration), infection came from the spleen. Some of the bacilli coming from the spleen could not be detained in the lungs and arrived directly at the lymph nodes without being previously phagocytosed by an alveolar macrophage. In the AER case, the main flow was from lungs to lymph nodes and then on to the spleen. Table 4 shows the flows between organs considered.

**Table 4:** *In silico* model of bacillary migration between organs, where BL_i_(t) is the bacillary load in spleen (i = s), lungs (i = l) and lymph nodes (i =  ln) at time t.

Bacilli flow	Mathematical expression
Spleen (s) to lungs (l)	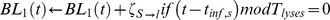
Spleen to lymph nodes (ln)	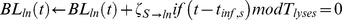
Lungs to lymph nodes	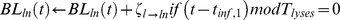
Lymph nodes to spleen	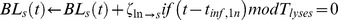

The auxiliary variables t_inf,s_, t_inf,l_ and t_inf,ln_ represent the time that spleen, lungs and lymph nodes have been infected. The parameters involved are detailed in *Table S1.*

Macrophage lysis can be considered to be a synchronic phenomenon at the beginning of the infection (i.e., infected macrophages burst approximately simultaneously), although it becomes desynchronized as time goes by. As such, bacillary migration should be considered to be discrete at the beginning of the infection and as continuous at some point thereafter. However, this phenomenon is only significant at the beginning of the infection as, when the bacillary load is high, the migratory effect between organs becomes irrelevant.

#### Trigger and dynamics of the specific IFN-γ response

The experimental results for the EV experiment show that the IR depends on the inoculated dose and, therefore, that it should also depend on the bacillary load: while BL remains small, no IR would be activated. Once a certain threshold is surpassed, an IR would be triggered. Another important experimental observation is that although very high inoculated doses (10^5^ CFU/mL) should be sufficient to activate the IR, IR measurements take some time to become positive. This suggests that, once the IR is triggered, there is some delay until it appears. These two observations are incorporated into the model using the parameters BL_thres_ and T_delay_, which represent the BL threshold to trigger the IR and the time delay for the detection of this response, respectively.

Once the IR is activated and generated in a lymphatic organ, lymphocytes are thought to enter the blood stream in order to reach the infected organs. As such, the model should consider the specific IR level in blood (IR_blood_) as an auxiliary variable. This variable increases with lymphocyte production at the rate γ, driven by BL_thres_ and T_delay_, and decreases due to lymphocyte lysis (with a rate ω) or when the lymphocytes are retained by an infected organ, as shown by Eq (2).
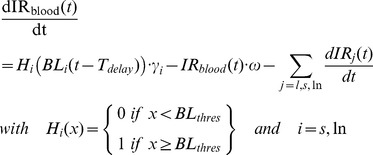
(2)


The retention of an IR by an infected organ is assumed to depend on the bacilli growing in that organ (i.e., it does not depend on the bacillary load of the organ, BL_i_(t), but on BL_i_(t)-a_IR_·IR_i_(t), as shown in Eq (1)). Other parameters involved in lymphocyte transport include the blood flow F_blood_ (volume of blood that enters the left ventricle of mouse heart per unit time) and the fraction of this flow that enters each of the organs considered (Q_i_ with i = l, s, ln). Eq (3) shows the equation for the model governing lymphocyte transport to these organs.
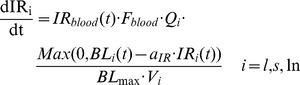
(3)


The model presented above for the dynamics of the IR is extremely simple but attempts to represent the overall dynamics of IR by taking into account the most relevant processes.

### Parameter Estimation

Parameters are those quantities that determine the result of the processes studied, for instance, the effective bacillary growth rate (µ). Their values must be directly extracted or estimated from experimental data and the literature or, in the worst case, inferred by fitting the model output to experimental results. Table 4 shows the list of parameters of the model, as well as their values and how these values have been fixed.

The duplication time was set to 24 hours, which is in accordance with the literature [22] and corresponds to a specific growth rate of 0.029 h^−1^. This value is compatible with the absolute maximum apparent growth rate observed experimentally, in particular in the EV experiment with low inoculum (10^2^ CFU/mL) in spleen. In these measurements, if we consider the interval between days 2 and 10 (Fig. 1) we obtain a duplication time of 1.39 days (R^2^ = 0.999). This value apparently shows a slower growth but is distorted by local factors such as bacillary flows. The parameters T_delay_ and BL_thres_ were inferred from experimental measurements, as detailed in Section 2.2. The extinction rate of specific lymphocytes (ω) was estimated using the expression ω =  ln2/t_1/2_, where t_1/2_ is the time at which the specific IFN-γ response is reduced to half of its maximum value. Reference [9] provides an order of magnitude of 3 hours for this value, which is in accordance with the half-life of specific lymphocytes in the absence of antigens. The percentage of blood that enters each organ, Q_i_, was chosen on the basis of the irrigation for each organ.

Finally, the remaining parameters were estimated by fitting the model simulation results to EV experimental results for inocula of 10^4^ and 10^5^ CFU/mL, only. To do so, an objective function was defined (Eq. 4):
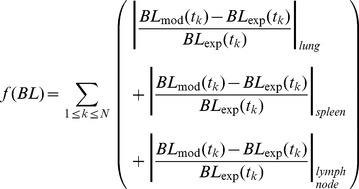
(4)


The model was implemented in the computing language C++ in order to solve those equations that had no analytical solution, which were solved using Euler's numerical method (controlling the instabilities). An exhaustive search of the set of parameters that minimized this objective function was performed in order to minimize differences between model output (mod) and experimental measurements (exp). To do so, two series of simulations of the mathematical model were performed, one for each initial concentration. Each series of simulations consisted of several repetitions with the same initial conditions, changing the random seed in order to reproduce experimental variability.

The initial bacillary load values were taken from experimental measurements by extrapolating data from day 2. These values were BL_l_ (0)  = 10 CFU, BL_s_ (0)  = 270 CFU and BL_ln_ (0)  = 1 CFU for the former and BL_l_ (0)  = 2500 CFU, BL_s_ (0)  = 11000 CFU and BL_ln_ (0)  = 10 CFU for the later. The outputs obtained from the model are shown in Figure 1. As can be observed, the initial increase of infection in several organs and the plateau once the specific IFN-γ response is activated are well reproduced. The best values for the parameters estimated are shown in Table 4.

### Model Validation

A second set of simulations was performed using the parameters estimated in the previous series. In this case only the initial conditions were modified in an attempt to reproduce AER and low concentration EV experiments without varying the model parameters. The initial infections were fixed as 10 bacilli in lungs (AER) and 5 bacilli in spleen (low concentration EV) with the same extrapolation method (see previous section). As shown in Figure 1, the model results were in agreement with experimental measurements. In the AER case, the coincidence between model and experimental results is high, although a slight underestimation of the plateau values can be observed. The low concentration EV simulations reproduce the infection evolution in spleen and the high variability of measurements in lungs and lymph nodes, which is also in agreement with experimental results.

The underestimation of the plateaus seen in most of the simulations suggests that although the model considers the effect of the specific IFN-γ response to be immediate, this might not be the case *in vivo* (as already suggested by the experimental results).

## Supporting Information

Table S1 **Parameters of the** ***in silico*** **model.** The Parameters of the *in silico* model described in Equations (1), (2) and (3) and in Table 3 have been included in this table, as well as their description, source and values.
(PDF)
