# Supplementary material for: To Achieve an Earlier IFN-γ Response Is Not Sufficient to Control Mycobacterium tuberculosis Infection in Mice
Source: PLoS One. 2014 Jun 24;9(6):e100830. doi: 10.1371/journal.pone.0100830 (PMC4069189; doi:10.1371/journal.pone.0100830)
Supplement: Table S1 — Parameters of the in silico model. The Parameters of the in silico model described in Equations (1), (2) and (3) and in Table 3 have been included in this table, as well as their description, source and values. (PDF) [file pone.0100830.s001.pdf]

Parameters of the *in silico* model described in Equations (1), (2) and (3) and in Table 3 as well as their description and values.

Parameter	Value	Units	Description	Source
μ	0.029	h^{-1}	Effective growth rate of bacilli inside mice	Bru&Cardona (2010) Experimental data (see text)
a_{IR}	500	bacilli per unit of IR	Bacilli that enter non replicative state because of the action of a lymphocyte	Fitted to experimental data
BL_{max}	2×10^9	bacilli/mL	Maximum bacterial load per unit of volume	Estimated from experimental data
V_l	0.50	mL	Effective volume of lungs	Estimated from Treuting et al 2012
V_s	0.75	mL	Effective volume of spleen	Estimated from Treuting et al 2012
V_{ln}	0.10	mL	Effective volume of lymph nodes	Estimated from Treuting et al 2012
$\zeta_{s\to l}$	$I_{0.4}\cdot N_{(0,10)}(5,2.5)^{a,b}$	bacilli	Bacilli that flow from spleen to lungs	Random distributed (normal distribution)
$\zeta_{s\to ln}$	$0.1\cdot I_{0.4}\cdot N_{(0,10)}(5,2.5)^{a,b}$	bacilli	Bacilli that flow from spleen to lymph node	Random distributed (normal distribution)
$\zeta_{l\to ln}$	$N_{(5,15)}(10,2.5)^{b}$	bacilli	Bacilli that flow from lungs to lymph node	Random distributed (normal distribution)
$\zeta_{ln\to s}$	$N_{(5,15)}(10,2.5)^{b}$	bacilli	Bacilli that flow from lymph node to spleen	Random distributed (normal distribution)
T_{lyses}	Random between 3 and 5	days	Time interval between the infection of a macrophage and its lyses	Lee *et al* 2006
T_{delay}	2.6	days	Time between activation of specific immune response in lymphatic organs and its	Deduced from experimental data (see text)
γ_s	100	IR units per hour	Production of specific lymphocytes in spleen during an hour	Fitted to experimental data
γ_{ln}	1000	IR units per hour	Production of specific lymphocytes in lymph node during an hour	Fitted to experimental data
ω	0.23	h^{-1}	Extinction rate of specific lymphocytes.	Bru and Cardona [2010]
BL_{thres}	3500	Bacilli	Minimum threshold of bacilli to trigger the specific immune response in lymphatic organs	Deduced from experimental data (see text)
F_{blood}	17	mL/min	Volume of blood that comes out from the left ventricle of mice heart in a minute	Estimated from Treuting et al 2012
Q_l	1.0	-	Fraction of blood that comes out from mice heart and enters lungs	Estimated (see text)
Q_s	0.30	-	Fraction of blood that comes out from mice heart and enters spleen	Estimated (see text)
Q_{ln}	0.01	-	Fraction of blood that comes out from mice heart and enters lymph node	Estimated (see text)

[a] I_q is a Bernoulli with parameter q.

[b] $N_{(m,n)}(\mu,\sigma)$ is a Normal around the mean μ and with deviation σ, truncated between m and n.
